# Supplementary material for: Gene module reconstruction elucidates cellular differentiation processes and the regulatory logic of specialized secretion
Source: bioRxiv. 2023 Dec 29:2023.12.29.573643. Preprint. [Version 1] doi: 10.1101/2023.12.29.573643 (PMC10793473; doi:10.1101/2023.12.29.573643)
Supplement: Supplement 8 [file NIHPP2023.12.29.573643V1-supplement-8.pdf]

bioRxiv preprint doi: https://doi.org/10.1101/2023.12.29.573643; this version posted December 29, 2023. The copyright holder for this preprint (which was not certified by peer review) is the author/funder. This article is a US Government work. It is not subject to copyright under 17 USC 105 and is also made available for use under a CC0 license.

Supplemental Tables

Table S1. Significantly enriched GO terms among notochord-enriched genes.

This table is provided as an Excel file.

Table S2. Significantly enriched GO terms among hatching gland-enriched genes.

This table is provided as an Excel file.

Table S3. List of gene modules in the notochord.

This table is provided as an Excel file. Genes with mentions in the main text are underscored. Modules are ordered alphabetically. Genes without module assignment are in the "Unassigned" group as the last row of the table.

Table S4. List of gene modules in the hatching gland.

This table is provided as an Excel file. Genes with mentions in the main text are underscored. Modules are ordered alphabetically. Genes without module assignment are in the "Unassigned" group as the last row of the table.

Table S5. Guide RNA sequences.

Target gene	crRNA sequence
tyr	CGTTGGGAAGGTCGGACACC
	TAACTTCACCATCCCGTACT
	GATGCATTATTACGTGTCCC
xbp1	GATTCAGACTCCACCACCTC
	GAGACTGGGGTTGGATACCT
	CACAAAGTCCTCCTGATATC
atf6	TGACACCAACCTCACTCATT
	GGCATAGTGACTACACGCGT
	ACCCATCCAGGTGTGCCCCA
creb3l1	TCAGATCCTCGAGATCCAAC
	CTGAGGGCACGTTGAGGAAC
	GGGCTCCTGCTTCACTAGAA
creb3l2	CGAATCACTTCCCACTGAGC
	GCCTGGATGTGTGGTGGCAC
	ACGAGAACCACGAGTTACGC
bhlha15	GGACGACCCAGACTTTACG
	CAAGATCGAGACTCTCACAT
	GGGTCATGGAGAACACTGAG

bioRxiv preprint doi: https://doi.org/10.1101/2023.12.29.573643; this version posted December 29, 2023. The copyright holder for this preprint (which was not certified by peer review) is the author/funder. This article is a US Government work. It is not subject to copyright under 17 USC 105 and is also made available for use under a CC0 license.

Table S6. Summary of the scRNA-seq datasets used in UPR TF target gene inference.

This table is provided as an Excel file.

Table S7. Lists of UPR TF target genes.

This table is provided as an Excel file. Only positive targets are included. Endogenous targets were inferred from the loss-of-function dataset in each cell type. Induced targets were inferred from the mis-expression dataset; genes induced in at least 2 cell types were included. The general secretion, ECM secretion, and gland-like secretion programs were defined in the main text (see section "*creb3l1*/*3l2* and *xbp1* regulate ECM secretion and gland-like secretion programs, respectively").
